# Complementary feeding practices in Northern Ghana: a mixed-methods study of prevalence, predictors, and socio-cultural influences

**DOI:** 10.1186/s40795-026-01398-x

**Published:** 2026-06-15

**Authors:** Abdul Razak Abubakari, Ahmad Sukerazu Alhassan, Abukari Salifu, Shamsu-Deen Ziblim, Suara Bakuri Sufyan

**Affiliations:** 1 School of Public Health, University for Development Studies, Tamale, Ghana; 2 School of Allied Health Sciences, University for Development Studies, Tamale, Ghana

**Keywords:** Socio-cultural, Children and infants, Dietary diversity, Meal frequency, Acceptable diet

## Abstract

**Background:**

Inappropriate complementary feeding (CF) practices contribute significantly to poor child development especially in malnutrition-burdened areas. While high knowledge and poor CF practices are widely documented, socio-cultural norms and taboos influencing child feeding practices have rarely been studied in context. This study examined the prevalence, predictors, and cultural beliefs influencing CF practices in Northern Ghana.

**Methods:**

A concurrent mixed-methods approach was employed. 326 mothers with children aged 6–23 months were recruited into the quantitative study while four Focus Group Discussions (FGDs) were held for the qualitative study. Statistical Package for Social Sciences (SPSS) version 21.0 was used to analyze the quantitative data (using both descriptive and inferential statistics) while the qualitative data was analyzed using thematic analysis.

**Results:**

About 66.6% of the children were timely initiated to CF (introduction of solid, semisolid or soft foods at 6–8 months), while 39.3%, 71.2%, and 37.1% met minimum dietary diversity (MDD), minimum meal frequency (MMF) and minimum acceptable diet (MAD), respectively. Appropriate complementary feeding (those who achieved timely initiation to CF, MDD, and MMF simultaneously) was met by 22.1%. Antenatal care (ANC) attendance and ethnicity were significant determinants of timely initiation of CF; children’s age and ethnicity were predictors of MAD; while household size and children’s age were associated with appropriate CF. In the qualitative results, early CF initiation (before 6th month of birth) was driven by perceived child readiness, and influence from mothers-in-law. Consumption of meat, eggs and dairy products were restricted by food taboos as well as spiritual and cultural beliefs. It was believed that consumption of these foods may cause delayed speech or immoral behavior in children. These beliefs restricted mothers’ willingness to provide animal-source foods.

**Conclusion:**

Appropriate complementary feeding remains sub-optimal despite high rate of timely CF initiation. These feeding practices are influenced by a wide range of maternal, household, and socio-cultural factors. These findings highlight the importance of considering socio-demographic and cultural influences when designing future child nutrition programs and interventions.

**Supplementary Information:**

The online version contains supplementary material available at 10.1186/s40795-026-01398-x.

## Introduction

Appropriate Infant and Young Child Feeding (IYCF) is fundamental for child’s health, growth and survival [1]. Optimal child feeding is a key component of the 2030 Agenda for Sustainable Development (Goal 2) and is connected to numerous Sustainable Development Goals (SDGs) as a practical measure to safeguard the survival and well-being of children and women [2]. As children advance in age to 6 months or more, breast milk alone becomes inadequate to meet their nutritional demands exposing them to malnutrition [3]. This underscores the need for the introduction of liquid/semi-solid/solid foods alongside breastfeeding as recommended by the World Health Organization (WHO) and United Nations Children’s Fund (UNICEF) [4]. For complementary feeding to achieve its purpose, it needs to be timely initiated, safe, adequate, and properly fed [5].

The recommended timing for initiating complementary feeding has sparked some scientific debate. While the WHO recommends complementary feeding initiation at six months, others such as Reilly and Wells argue that delaying complementary feeding until exactly six months may not be appropriate for all infants, especially where growth and energy demands increase earlier than expected [6]. Nonetheless, the WHO continues to recommend the initiation of complementary feeding at six months because early introduction may expose infants to infections, gastrointestinal disturbances, and poor breastfeeding outcomes. Beyond just the timing, the nutritional quality of complementary foods is equally important to ensure optimal growth and development of the child. Thus, complementary foods should provide adequate energy, protein, and essential micronutrients especially at age 6–23 months when children’s nutritional demands increase rapidly. The WHO IYCF indicators such as MDD, MMF, and MAD are important measures used in assessing the adequacy of complementary feeding practices [4].

On a global scale, complementary feeding is proven to be suboptimal especially in West and Central Africa [7]. In Ghana, where almost a third of young children are fed with unhealthy foods (commercially prepared snacks, sugar-sweetened beverages, fried foods, or foods high in sugar, salt, or unhealthy fats) [8], child malnutrition remains unacceptably high with the worst rates of stunting (30%) and wasting (8%) recorded in the Northern Region [8]. Within this region, the Yendi Municipality stands out for recording one of the highest rates of malnutrition with over 27% underweight, 38% stunted and 21% wasted children [9]. Despite this alarming situation, there is limited empirical evidence on complementary feeding practices and their determinants.

This study explores how various individual, household, and healthcare related factors operate to influence child feeding practices. For instance, maternal age, occupation, marital status, educational level, and ethnicity were included in this study because they are reported to often influence how caregivers practice appropriate complementary feeding [10, 11]. Moreover, household size was considered because it can potentially affect the availability of food in the household as well as quality of care given to young children [12]. Utilization of healthcare services such as ANC are also taken into consideration as it exposes mothers to nutrition counseling opportunities which may shape child feeding practices [13, 14]. Previous studies have also found child related factors including age, sex, birth index, place of birth, and mode of delivery as significant determinants of complementary feeding practices [13–15].

While some studies in Ghana have investigated complementary feeding at the regional and national levels, there is a lack of in-depth understanding on how cultural, spiritual and household factors shape child feeding practices particularly in high malnutrition burden settings like the Yendi Municipality. Hence, this study adopted a mixed-method approach to investigate the prevalence and predictors of complementary feeding as well as socio-cultural norms and taboos influencing child feeding practices in the Yendi Municipality. This approach offers a novel contribution to the literature by combining both statistical data and thematic insights to offer comprehensive understanding on culturally driven child feeding behaviors which is often neglected in key nutrition interventions and programs.

## Methods

### Study setting

The study was conducted in the Yendi Municipality, situated in the Eastern Corridor of Northern Ghana between Latitudes 90–350 North, 00-300 West, and 00-150 East. It has an average household size of 5.3, dominated by children and headed by men. Also, it is made up of different ethnic groups, primarily Dagombas and Konkombas. About half of the population lives in rural areas and agriculture is the dominant occupation with about 15% of its arable land under cultivation [16].

### Study design

The study adopted concurrent mixed-method approach, which involves the collection, merging and use of both quantitative and qualitative data in a single study simultaneously. Compared to studies that do not combine both quantitative and qualitative approaches, a mixed-method approach provides a deeper, and broader understanding of research problems [17]. The quantitative method measured the magnitude of various child feeding parameters while the qualitative method examined sociocultural factors that have direct or indirect impact on child feeding practices.

### Sampling methods

Mothers aged 15–49 years with children aged 6–23 months residing in the Yendi Municipality were eligible to participate in the study. However, children with a condition that may compromise the child’s dietary intake and those on specific diet therapy or had been restricted from consuming a variety of foods by a nutritionist or other healthcare practitioners were exempted from the study.

For the quantitative data, 326 mothers with children aged 6–23 months were recruited from 20 communities through multistage sampling technique. The first stage adopted the existing Ghana Health Service clusters/sub-districts. In the second stage, a list of all communities in each cluster were compiled and five communities were selected from each cluster through simple random sampling while ensuring adequate geographical coverage. A sampling frame was formed in each selected community by enumerating all households with children aged 6–23 months old with the assistance of trained community health volunteers. This list provided the basis for determining the number of interviews to be conducted in each community using probability proportional to size. Eligible households were then selected using systematic sampling. In a household with more than one eligible subject or if a caregiver has more than one child who satisfies the criteria, simple random sampling was used to select one.

For the qualitative data, purposive sampling was used to recruit mothers for FGDs. Community health volunteers assisted in identifying eligible mothers who were willing to share their experiences and perceptions regarding complementary feeding practices. In all, four FGDs were conducted across four different sub-districts of the Municipality to ensure diversity of perspectives. This number was determined by data saturation, a point where no new themes or insights were emerging from additional discussions. After four FGDs, thematic redundancy was observed and hence, additional discussions were deemed unnecessary. The discussions were held at Nayili Fong, Adibo, Naa lo’u and Kpalgigbini. Each group composed of eight mothers, a note taker and a facilitator (the corresponding author) which is consistent with qualitative research recommendations [18, 19]. None of the eligible mothers declined participation, and there were no dropouts after recruitment.

### Data collection instruments

The quantitative data were collected using structured questionnaire which was adopted from the WHO IYCF indicators and previous complementary feeding studies [15, 20] (provided as Supplementary File 1). The questionnaire included sections on socio-demographic characteristics as well as complementary feeding practices such as the introduction of solid/semisolid/soft foods at 6–8 months. Dietary diversity and meal frequency were also measured using 24-hour dietary recall. The questionnaire was pretested among 20 women in a similar population and refined for clarity.

For the qualitative component, a semi-structured FGD guide was employed to explore mothers’ perceptions, beliefs, and experiences regarding complementary feeding. The guide was adopted and modified from previous literature [21, 22] (provided as Supplementary File 2) and pretested with two focus groups who share similar characteristics with the study respondents. The guide covered topics such as timing of introduction of solid/semisolid/soft foods and cultural influences on complementary feeding practices.

### Data collection methods

Data collection was conducted from August 23 to September 15, 2024. The quantitative data was collected by the researchers and trained research assistants with prior experience in data collection through face-to-face interviews using structured questionnaire. The data were captured electronically using mobile phones and tablets equipped with Kobo Toolbox to ensure real-time data entry and reduced data entry errors. The questionnaire assessed respondents’ socio-demographic characteristics and complementary feeding practices including the period of introducing solid, semi-solid, or soft foods and 24-hour dietary recall for their respective children. Questions were asked by the data enumerators and responses were given by the mothers. For mothers who do not understand English Language, the researchers interpreted it to them in their local dialect (mostly Dagbani and Konkomba), and their responses were ticked against the applicable response/option on the questionnaire. Scenarios were given to emphasize clarity and to gain reliable responses from mothers especially those who showed poor understanding of the interview questions. Although respondents and children’s socio-demographic data such as age, number of ANC visits, mode of delivery and place of delivery were given orally by the mothers, these variables were confirmed using the ANC attendance book. Overall, it took approximately 30 min to complete one questionnaire.

Regarding the qualitative data, a serene environment was arranged for the FGDs at the community level. At Nayili Fong, Adibo, and Naa lo’u, the interviews were conducted in Dagbani Language while in Kpalgigbini, it was done in Konkomba Language. The discussions were moderated by the corresponding author who has training in qualitative research and maternal and child health. Participants were informed about the study purpose, and no prior relationship existed between the researcher and participants before data collection. Audio recordings and field notes were used to capture data, including non-verbal cues. Averagely, each FGD lasted for 1 h 30 min.

### Study variables and assessment

The dependent variables for this study were the WHO IYCF indicators: introduction of solid/semisolid/soft foods at 6–8 months, MDD, MMF, and MAD. Timely initiation of complementary feeding (TICF) was assessed based on the age of the child (in months) at which solid/semisolid/soft foods were introduced. Thus, children who were introduced to solid/semisolid/soft foods at age 6–8 months were declared to have been timely introduced to complementary foods. Also, the criterion for meeting MDD was the consumption of foods from at least 5 out of the 8 food groups within a 24-hour period preceding the survey time [23]. The reference food groups were: breast milk; roots, tubers and grains; nuts and legumes; dairy products (milk, infant formula, yogurt, cheese); fleshy foods (fish, poultry, meat, organ meats); eggs; vitamin-A rich fruits and vegetables; and other fruits and vegetables [24]. Depending on whether the child is breastfed or not, MMF is taken as the total number of times a child eats solid or semi-solid foods within 24-hours, including meals and snacks. The MMF scores were computed in accordance with the WHO guidelines regarding the amount of daily meals that young children in different age groups are supposed to receive in addition to breastfeeding [23]. Thus, a breastfed child was declared to have met the MMF score if he/she was fed solid, semi-solid or soft foods 2 to 3 times/day for those aged 6–8 months and 3 to 4 times/day for those aged 9–23 months. Also, a non-breastfed child was reported to have met this score if he/she ate 4 times or more/day for those aged 6–23 months. The MAD was assessed by computing the proportion of children who have met the requirements for both MDD and MMF within the same 24-hour period in both non-breastfed and breastfed children.

Most previous studies often focus on the TICF, MMF, and MDD as separate outcomes which may overlook whether children simultaneously receive age-appropriate dietary initiation, dietary diversity, and meal frequency. In this study, we constructed a composite indicator called appropriate complementary feeding (ACF), defined as meeting TICF, MMF, and MDD. This composite indicator provides a broader measure of optimal feeding practices by identifying children who meet all key the WHO recommended feeding components concurrently. This approach has been used in similar nutritional epidemiological research to reflect holistic feeding adequacy [25]. That notwithstanding, this should be interpreted as an aggregate indicator rather than a formal WHO indicator.

Socio-demographic characteristics of mothers (age, ethnicity, religion, marital status, educational level, occupation, household size, and number of ANC visits) and that of their children (age, gender, place and mode of delivery as well as birth index) were the independent variables in this study. Tolerance and variance inflation factor (VIF) were used to check for multicollinearity among the independent variables. All the variables recorded a tolerance level above 0.10 (range: 0.400–0.989) and a VIF values below 5 (range: 1.011–2.497) which indicates the absence of multicollinearity among the predictor variables.

### Data analysis

The data was analyzed using SPSS (version-21) software. Means and standard deviations were calculated for continuous variables while percentages and frequencies were computed for categorical variables. In the bivariate analysis, associations between outcome and predicting variables were examined using unadjusted logistic regression model with significance level set at p-value ≤ 0.200. Multivariable binary logistic regression analysis was run to ascertain the independent effects of explanatory variables on the outcome variables by controlling for possible confounders. The variables included in the model were selected based on theoretical relevance and significance level at the bivariate analysis. Declaration of significance in the adjusted model was considered at p-value ≤ 0.050. Although descriptive statistics were reported for all five outcome variables, logistic regression was fitted only for the TICF, MAD, and ACF. However, MDD and MMF were not modeled separately because they are direct component indicators of MAD, which is a composite measure reflecting both dietary diversity and meal frequency.

The qualitative data was analyzed manually using thematic analysis and following principles outlined by Creswell [26] and Braun and Clarke [27]. Firstly, the audio recordings from the FGDs were transcribed verbatim and cross-checked against the recordings by two independent members of the research team to ensure accuracy. Secondly, the researchers familiarized themselves with the transcripts by repeatedly reading them. Statements that were related or comparable were identified and coded independently by two members of the team (3rd and 4th authors) after which the codes were discussed among the entire research team to address any discrepancies. Similar and related codes were grouped together to create subthemes and related sub-themes were further merged to create major themes. The analyses were conducted manually without the use of any qualitative data management software. Also, participant validation of findings was not conducted due to logistical constraints.

## Results

### Socio-demographic characteristics of respondents

A total of 326 mothers were recruited into the study of which many of them 128 (39.3%) were between 25 and 34 years and the mean (± standard deviation) maternal age was 29.85 (± 7.045) years. Almost all the respondents (98.2%) were married and over half of them (58.0%) had no formal education. Farming was their major (59.8%) occupation and 71.5% belonged to the Dagomba ethnic group. Over two-thirds (74.9%) of them belonged to the Islamic faith and 77.4% were living in households with more than 8 people. Also, the proportion of the respondents who had 4 to 7 ANC visits constituted the majority with 64.1%. Regarding children’s characteristics, the highest proportion (38.3%) of them were between 6 and 11 months of age with a mean (± SD) age of 13.91 (± 5.474) months. Also, over half (56.1%) of them were males and a significant majority (81.3%) were born in a health facility. Almost all (96.3%) of them were born through Spontaneous Vaginal Delivery (SVD) and those who were their mother’s fourth born or higher constituted the biggest proportion (38.0%) (see Table 1).

**Table 1:** Characteristics of respondents

Maternal characteristics			Children’s characteristics		
Variable	Frequency (*n*)	Percentage (%)	Variable	Frequency (*n*)	Percentage (%)
Age (years)*			Age (months)**		
15 to 24	104	31.9	6 to 11	125	38.3
25 to 34	128	39.3	12 to 17	96	29.4
35 to 49	94	28.8	18 to 23	105	32.3
Marital status			Sex		
Unmarried	6	1.8	Male	183	56.1
Married	320	98.2	Female	143	43.9
Educational level			Place of birth		
No formal education	189	58.0	Home	61	18.7
Primary/JHS/JSS	85	26.0	Health facility	265	81.3
SHS/SSS/Tertiary	52	16.0	Mode of delivery		
Occupation			CS	12	3.7
Unemployed	16	4.9	SVD	314	96.3
Farming	195	59.8	Birth index/order		
Trading	66	20.2	1st born	85	26.1
Trained/Skilled/vocational	49	15.0	2nd born	60	18.4
Ethnicity			3rd born	57	17.5
Dagomba	233	71.5	4th born and above	124	38.0
Konkomba/others	93	28.5			
Household size (people)					
1 to 4	10	3.1			
5 to 8	65	19.9			
More than 8	251	77.0			
Number of ANC visits					
Less than 4 visits	54	16.6			
4 to 7 visits	209	64.1			
At least 8 visits	63	19.3			

* Mean maternal age [± standard deviation (SD)] was 29.85 ± 7.05 years

**Mean child age [± standard deviation (SD)] was 13.91 ± 5.474 months

### Prevalence of complementary feeding indicators

Majority (66.6%) of the children in this study were timely introduced to complementary feeding (TICF). MDD, MMF and MAD were achieved by 39.3%, 71.2% and 37.1% of the children respectively while a little over a fifth (22.1%) met the requirements for age-appropriate complementary feeding practices (see Table 2).

**Table 2:** Prevalence of complementary feeding indicators

Variable	Frequency (*n*)	Percentage (%)
Timely initiation of complementary feeding		
Met	217	66.6
Not met	109	33.4
Minimum Dietary Diversity (MDD)		
Met	128	39.3
Not met	198	60.7
Minimum Meal Frequency (MMF)		
Met	232	71.2
Not met	94	28.8
Minimum Acceptable Diet (MAD)		
Met	121	37.1
Not met	205	62.9
Complementary feeding		
Appropriate	72	22.1
Not appropriate	254	77.9

### Factors associated with complementary feeding initiation

In the bivariate model, occupation, ethnicity, household size, and ANC attendance were revealed to be significantly associated with timely introduction of solid/semisolid/soft foods. Meanwhile, at the multivariate level using binary logistic regression model, ANC attendance and ethnicity remained statistically significant factors associated with timely initiation of complementary feeding. For example, mothers who had eight or more ANC attendance were over two times at higher odds of timely initiating complementary feeding [Adjusted Odds Ratio (AOR): 2.35, 95% Confidence Interval (CI): 1.05–5.26, p-value: 0.038] compared to their counterparts who had less than four visits. Also, children born to mothers from Konkomba and/or other tribes were over four times more likely to be timely introduced to complementary foods [AOR: 4.10, 95% CI: 2.09–8.03, p-value: < 0.001] compared to their counterparts born to Dagomba mothers (see Table 3).

**Table 3:** Factors associated with TICF

Variable	COR (95% CI)	*p*-value	AOR (95% CI)	*p*-value
Maternal Age (years)				
15 to 24	Ref			
25 to 34	1.10 (0.63–1.93)	0.737		
35 to 49	0.69 (0.38–1.23)	0.204		
Marital status				
Married	Ref			
Unmarried	0.10 (0.08–5.52)	0.996		
Educational level				
No formal education	Ref			
Basic education	0.79 (0.46–1.35)	0.385		
Secondary/Tertiary education	1.07 (0.55–2.08)	0.837		
Occupation				
Unemployed	Ref			
Farming	2.05 (0.74–5.70)	0.171	1.41 (0.48–4.19)	0.535
Trading	1.75 (0.58–5.26)	0.319	1.83 (0.59–5.74)	0.297
Trained/Skilled/vocational	2.77 (0.86–8.90)	0.087	2.09 (0.62–7.02)	0.233
Ethnicity				
Dagomba	Ref		Ref	
Konkomba/others	3.52 (1.91–6.48)	< 0.001	4.10 (2.09–8.03)	< 0.001
Household size (people)				
1 to 4	Ref		Ref	
5 to 8	0.13 (0.02–1.08)	0.059	0.14 (0.02–1.25)	0.079
More than 8	0.25 (0.03–1.98)	0.188	0.25 (0.03–2.09)	0.201
Number of ANC visits				
Less than 4 visits	Ref		Ref	
4 to 7 visits	2.74 (1.48–5.05)	0.001	2.73 (1.41–5.28)	0.003
At least 8 visits	2.01 (0.95–4.22)	0.066	2.35 (1.05–5.26)	0.038
Children’s Age (months)				
6 to 11	Ref			
12 to 17	1.27 (0.72–2.26)	0.409		
18 to 23	0.93 (0.54–1.59)	0.777		
Sex				
Male	Ref			
Female	1.31 (0.82–2.09)	0.255		
Place of birth				
Health facility	Ref			
Home	0.87 (0.48–1.55)	0.629		
Mode of delivery				
Spontaneous Vaginal Delivery (SVD)	Ref			
Caesarean Section (CS)	0.69 (0.22–2.24)	0.540		
Birth index/order				
1st born	Ref			
2nd born	0.80 (0.40–1.61)	0.54		
3rd born	1.10 (0.53–2.27)	0.81		
4th born and above	0.88 (0.49–1.58)	0.66		

*COR *Crude Odds Ratio, *AOR *Adjusted Odds Ratio, *CI* Confidence Interval

### Factors associated with minimum acceptable diet

The unadjusted model reveals maternal age, children’s age, ethnicity, household size, number of ANC visits, and mode of delivery as the variables significantly associated with the MAD score of the children. At the multivariate level, where possible confounders were controlled, children’s age and ethnicity were the only covariates that showed statistical significance with children’s MAD scores. Higher children’s age (18 to 23 months) was linked with more than three folds increased odds of meeting MAD [AOR: 3.55, 95% CI: 1.94–6.48, p-value: < 0.001] in comparison with their counterparts aged 6–11 months. Moreover, children of Dagomba mothers had higher odds of meeting MAD compared to children of mothers from Konkomba or other ethnic groups [AOR: 0.47, 95% CI: 0.26–0.84, p-value: 0.003] (see Table 4).

**Table 4:** Factors associated with children’s MAD scores

Variable	COR (95%CI)	*p*-value	AOR (95%CI)	*p*-value
Maternal Age (years)				
15 to 24	Ref			
25 to 34	0.90 (0.52–1.56)	0.697	0.88 (0.49–1.58)	0.670
35 to 49	1.89 (1.07–3.35)	0.030	1.83 (0.98–3.40)	0.058
Marital status				
Married	Ref			
Unmarried	3.00 (0.35–25.99)	0.319		
Educational level				
No formal education	Ref			
Basic education	1.10 (0.65–1.87)	0.714		
Secondary/Tertiary education	1.00 (0.53–1.89)	0.997		
Occupation				
Unemployed	Ref			
Farming	0.89 (0.31–2.56)	0.832		
Trading	1.57 (0.51–4.81)	0.431		
Trained/Skilled/vocational	0.74 (0.23–2.39)	0.610		
Ethnicity				
Dagomba	Ref			
Konkomba/others	0.42 (0.24–0.72)	0.002	0.47 (0.26–0.84)	0.011
Household size (people)				
1 to 4	Ref			
5 to 8	0.39 (0.10–1.52)	0.176	0.34 (0.08–1.50)	0.154
More than 8	0.38 (0.10–1.38)	0.141	0.40 (0.10–1.63)	0.201
Number of ANC visits				
Less than 4 visits	Ref			
4 to 7 visits	0.63 (0.34–1.16)	0.136	0.66 (0.34–1.27)	0.214
At least 8 visits	0.94 (0.45–1.95)	0.863	1.14 (0.50–2.58)	0.759
Children’s Age (months)				
6 to 11	Ref			
12 to 17	2.08 (1.16–3.72)	0.014	2.01 (1.08–3.72)	0.027
18 to 23	3.64 (2.07–6.41)	< 0.001	3.55 (1.94–6.48)	< 0.001
Sex				
Male	Ref			
Female	1.17 (0.74–1.84)	0.500		
Place of birth				
Health facility	Ref			
Home	1.03 (0.58–1.83)	0.916		
Mode of delivery				
Spontaneous Vaginal Delivery (SVD)	Ref			
Caesarean Section (CS)	2.46 (0.76–7.92)	0.132	1.98 (0.58–6.82)	0.279
Birth index/order				
1st born	Ref			
2nd born	0.83 (0.41–1.66)	0.594		
3rd born	0.83 (0.41–1.68)	0.600		
4th born and above	1.12 (0.64–1.97)	0.697		

*COR* Crude Odds Ratio, *AOR *Adjusted Odds Ratio, *CI *Confidence Interval

### Factors associated with appropriate complementary feeding practices

The bivariate model revealed maternal age, occupation, ethnicity, household size, number of ANC visits, children’s age, and their sex to be significantly associated with appropriate complementary feeding practices but in the adjusted model, household size and children’s age remained significant. For instance, children from bigger households (5–8 people) had about 80% lesser odds [AOR: 0.20, 95% CI: 0.04–0.93, p-value: 0.040] of having appropriate complementary feeding compared to their colleagues in smaller households (1–4 people). In addition, the odds of appropriate complementary feeding among older children (18–23 months) was more than two folds [AOR: 2.42, 95% CI: 1.21–4.84, p-value: 0.013] compared to their younger counterparts aged 6–11 months (see Table 5).

**Table 5:** Factors associated with appropriate complementary feeding practices

Variable	COR (95% CI)	*p*-value	AOR (95% CI)	*p*-value
Maternal Age (years)				
15 to 24	Ref			
25 to 34	1.46 (0.76–2.81)	0.253	1.50 (0.75–2.99)	0.254
35 to 49	1.64 (0.82–3.26)	0.160	1.61 (0.75–3.44)	0.218
Marital status				
Married	Ref			
Unmarried	1.43 (0.16–12.40)	0.748		
Educational level				
No formal education	Ref			
Basic education	1.14 (0.61–2.13)	0.672		
Secondary/Tertiary education	1.61 (0.80–3.24)	0.180		
Occupation				
Unemployed	Ref			
Farming	0.50 (0.16–1.52)	0.222	0.56 (0.16–2.03)	0.379
Trading	1.18 (0.36–3.80)	0.786	1.26 (0.34–4.63)	0.730
Trained/Skilled/vocational	0.43 (0.12–1.58)	0.202	0.41 (0.10–1.70)	0.217
Ethnicity				
Dagomba	Ref			
Konkomba/others	0.59 (0.32–1.11)	0.104	0.91 (0.44–1.88)	0.804
Household size (people)				
1 to 4	Ref			
5 to 8	0.18 (0.04–0.75)	0.018	0.20 (0.04–0.93)	0.040
More than 8	0.29 (0.08–1.05)	0.060	0.34 (0.08–1.39)	0.133
Number of ANC visits				
Less than 4 visits	Ref			
4 to 7 visits	1.62 (0.72–3.68)	0.247	1.90 (0.79–4.59)	0.153
At least 8 visits	2.30 (0.91–5.82)	0.079	2.75 (0.99–7.65)	0.053
Children’s Age (months)				
6 to 11	Ref			
12 to 17	1.76 (0.89–3.46)	0.102	1.75 (0.85–3.62)	0.128
18 to 23	2.23 (1.17–4.26)	0.015	2.42 (1.21–4.84)	0.013
Sex				
Male	Ref			
Female	1.48 (0.87–2.50)	0.146	1.47 (0.84–2.56)	0.179
Place of birth				
Health facility	Ref			
Home	0.84 (0.42–1.67)	0.615		
Mode of delivery				
Spontaneous Vaginal Delivery (SVD)	Ref			
Caesarean Section (CS)	2.63 (0.81–8.56)	0.107	2.94 (0.84–10.25)	0.092
Birth index/order				
1st born	Ref			
2nd born	0.61 (0.26–1.47)	0.272		
3rd born	1.24 (0.57–2.71)	0.588		
4th born and above	1.06 (0.55–2.05)	0.861		

*COR *Crude Odds Ratio, *AOR *Adjusted Odds Ratio, *CI *Confidence Interval

### Socio-cultural beliefs influencing complementary feeding practices

#### Sub-theme 1: beliefs about complementary feeding initiation

##### When to initiate complementary feeding

Although the results from the quantitative survey indicate that approximately two-thirds of mothers reported initiating complementary feeding at 6–8 months, the qualitative narratives revealed persistent beliefs and social pressures that support poor initiation. A section of the mothers reported initiating complementary feeding before the WHO recommended 6-8 month period. This practice was largely influenced by mothers-in-law where children’s behaviors were used to influence the timing of complementary feeding initiation. For instance, early interest in family foods by the child was perceived as the right time to initiate complementary feeding. A 28-year-old respondent explained:

*“Just as adults are different*,* so are children. Some of them show interest in eating as early as 3 months of age. So*,* for me*,* I think they should be given food whenever they show the urge*,* irrespective of their age”.*

Also, some respondents interpreted a child’s gripping of food to mean breast milk alone was insufficient for them. A 27-year-old respondent narrated:

*“At 4 months old*,* she used to crawl to her siblings whenever they were eating*,* trying to grab the food. My mother-in-law said the child’s actions were signifying that the breast milk was no longer enough for her. That was when I started giving her porridge”.*

##### Perceived benefits of initiating complementary feeding early

Early initiation of complementary feeding (before six months) was reported to facilitate children’s growth and development. Some of the respondents were of the view that initiating complementary feeding earlier makes children gain weight and become strong. According to a 37-year-old mother:

*“It is very good to start complementary feeding early*,* maybe around 4 months. Let me tell you my experience*,* when I started giving my baby porridge at four months*,* she became more active and gained weight quickly. At a point*,* she even looked older than other children who were 5 or 6 months old. Whenever I take the child to the welfare clinic*,* the nurses praise me for how the baby has gained weight”.*

Another commonly held belief was that once a child begins to sit independently, their energy needs increase beyond what milk can provide. Mothers felt that complementary feeding was necessary to meet these energy demands. One 35-year-old respondent narrated:

“What happens is that, when a child starts sitting, their energy demands increase and breast milk is not enough to provide that energy. So, at that point there is the need to start complementary feeding”.

##### Perceived consequences for initiating CF late

Others mentioned that delay in initiating complementary feeding makes children hate food or develop eating disorders when they grow up. One 33-year-old respondent said:

*“Although the nurses often tell us to initiate complementary feeding at 6 months*,* we do not always adhere. Waiting till 6 months to initiate complementary feeding causes eating disorders among children when they grow up. For me*,* I always ensure that my children are given family foods when they are 3 months*,* so that they grow up to love food”.*

To buttress this assertion, a 30-year-old participant added:

*“Waiting until the child is six months to start complementary feeding is not best for the child. From my experience in childbirth*,* such children often look weak and easily fall sick”.*

#### Sub-theme 2: Misconceptions against the consumption of certain foods

##### Taboos surrounding meat and eggs

Participants described some misconceptions that forbid children from consuming certain foods such as meat and eggs. These foods were believed to cause deviant behaviors in children. One 37-year-old respondent explains:

*“Our customs forbid children from eating meat or eggs. Elders have observed this for a long time and they have found that these foods make children grow up as beggars”.*

Another 29-year-old respondent elaborated on this belief explaining that:

*“You know meat and eggs are expensive in this setting. So*,* children who form the habit of eating these foods are likely to steal money to buy them which will make them become thieves in future”.*

##### Perceptions of bad omens associated with dairy products

Dairy products during complementary feeding were strongly rejected by some respondents with the notion that they bring bad omens to the family. One mother narrated that children who are given dairy foods such as cow milk are likely to attract bad omen into their family.

*“When you feed your child with cow milk (for instance)*,* it signifies that the child’s mother is not alive. This can eventually invite a bad omen into the family” (41-year-old mother).*

Other respondents follow commandments of spiritualists to deny their children certain animal products. One 26-year-old respondent narrates:

*“I and my husband were married for 5 years without a child and so he (my husband) consulted a spiritualist who made some sacrifices leading to me picking a seed and eventually giving birth to my present child. So*,* the spiritualist said it was forbidden for the child to consume any meat or product coming from these animals (goat and fowls) else*,* a bad omen will befall the family”.*

## Discussion

### Prevalence of complementary feeding practices

In this study, about two-thirds (66.6%) of children were timely initiated to complementary foods which is consistent with reports in Ethiopia [11] but higher than studies in Bangladesh [28] and Pakistan [13]. Also, our result is lower than the 72.3% in Nigeria [14]. The qualitative findings provide a deeper insight, revealing that while many mothers timely initiated CF, some started giving foods before 6 months. The early initiation of CF (before 6 months) was influenced by a wide range of cultural factors including the belief that it makes children strong and helps them gain weight, confirming prior studies in Ghana [29, 30]. Also, children’s gestures such as grabbing of food items were interpreted as signals to start CF aligning with a previous literature [31]. Since poor CF initiation correlates with increased incidence of stunting and wasting, this pattern may contribute to poor nutritional outcomes among children in the setting and similar contexts [32, 33].

Beyond just initiation, 39.3% of the children received diversified diets, 71.2% achieved MMF, and 37.1% met MAD. These figures are higher than studies in Northern Ghana [34], and Nigeria [14] but lower than reports from South Africa [35] and Indonesia [36]. Additionally, although 22.1% of the children in this study had appropriate complementary feeding which is higher than prior studies [14, 25], this figure remains suboptimal. This may be explained by the fact that the study was conducted in a predominantly rural setting where access to nutritional information and health services are limited. This therefore highlights the persistent gaps in child feeding practices despite several local and international efforts.

A striking insight from the findings is that although many children met MMF, their diets were mostly not diversified. The qualitative findings provide important context for these quantitative results. While the survey quantified the extent of suboptimal feeding practices, the interviews revealed that cultural beliefs and household influences significantly shaped caregivers’ food choices for children. For instance, children were restricted from eating meat and eggs with the belief that such foods promote theft or begging among children later in life, which aligns with reports from Nigeria [37]. These qualitative narratives regarding food restrictions help explain the low prevalence of MDD observed in the quantitative findings. Indeed, animal-source foods such as meat and eggs are generally expensive in many places and hence the argument is that if children are brought up on such expensive foods, they may eventually have to steal in order to sustain their eating habits when they grow up. Similar prohibitions have been reported elsewhere in Ethiopia [38], Nigeria [39], and Kenya [40]. These studies have also documented a strong opposition regarding the intake of meat and eggs by children which were believed to be indigestible and impure for children’s consumption respectively. Although these studies were conducted in different settings, the convergence of their findings can be attributed to factors such as acculturation and cultural assimilation. This also suggests that food taboos remain important barriers to dietary adequacy across different settings.

### Determinants of complementary feeding practices

We found that mothers with higher ANC attendance (≥ 4 visits) had higher odds of timely initiating CF than their counterparts with fewer ANC visits which resonates with other studies [41, 42]. Probably, mothers who attend ANC receive nutrition education and its associated benefits, underscoring the need to strengthen maternal health services. Although higher ANC attendance was associated with TICF quantitatively, the qualitative findings suggest that household influences, particularly from mothers-in-law, may sometimes override professional nutrition counseling by encouraging the introduction of complementary foods before six months. This demonstrates a potential conflict between biomedical knowledge and cultural preference, suggesting that future nutrition programs may benefit from considering the broader family context in child feeding counselling.

Ethnicity was also associated with child feeding practices with mothers in minority tribes (Konkombas and others) having higher odds of timely initiating complementary feeding but lower odds of meeting MAD compared to Dagomba mothers (majority tribe). This pattern suggests that ethnic variations in child-feeding behaviors may exist within the study setting, potentially reflecting differences in cultural beliefs and food preferences. Similar associations have been reported in other African countries where ethnicity influences complementary feeding practices through culturally shaped food choices and household food production systems [43, 44]. In addition, ethnic/cultural factors often dictate the choice of foods by caregivers and therefore children may be influenced to consume foods that are only in line with their caregivers’ ethnic values [45]. Although the qualitative interviews were not designed to compare ethnic groups, they however revealed several culturally embedded food restrictions relating to meat, eggs, and dairy products, which may help explain the quantitative differences observed. Nonetheless, because this study did not directly measure specific cultural practices or agro-ecological mechanisms underlying these differences, these interpretations should be viewed as plausible explanations rather than confirmed causal pathways.

Furthermore, older children (18–23 months) had higher odds of meeting MAD and ACF than their younger ones (6–11 months), which corroborates findings from studies in sub-Saharan Africa [46] and Asia [47], but contradicts evidence from West Africa [48] and Pakistan [49]. Similar patterns have been noticed between dietary diversity and acceptable diet across multiple studies, including the present one. Children often perform poorly in these indicators partly due to certain societal beliefs that restrict their consumption of animal proteins and fruits [50, 51]. Some authors also argue that younger children find it strange when introduced to complementary foods making it difficult for them to meet age-appropriate dietary adequacy [52]. This is concerning given that the first 12 months are critical periods for active growth and brain development, and poor feeding practices at this stage may contribute to irreversible growth faltering [53], including stunting [54] and wasting [55]. These findings, therefore, suggest that younger children may benefit from greater attention in future nutrition programs aimed at promoting age-appropriate complementary feeding practices.

Another trend observed in this study was that, children in smaller households had better odds of receiving appropriate complementary foods compared to their peers in bigger households which agrees with an Ethiopian study [56]. Unlike smaller households, bigger households are more likely to face food insecurity due to high consumption demands [57] which may compromise children’s dietary intake. This could explain why children in bigger households failed to meet the requirements for age-appropriate complementary feeding practice in the present study. A second possible explanation is that mothers in smaller households may have adequate time to prepare age-appropriate meals for their children unlike the situation in larger households where children are made to consume regular family foods due to heavy workload on their caregivers.

### Program and policy implications

The relatively high rates of timely initiation of solid, semi-solid, or soft foods compared to the low rate of dietary diversity suggests significant gaps in appropriate child feeding practices in the setting. These findings suggest the potential value of strengthening community-based nutrition programs to focus on improving dietary diversity through the use of locally-available and affordable foods. Considering the strong influence of ethnic and cultural beliefs on child feeding practices, it is also crucial to integrate culturally sensitive interventions and behavior change communication strategies that will address food taboos and encourage the consumption of nutrient-dense meals by children. In addition, policy makers may consider placing more emphasis on younger children by focusing on age-appropriate complementary feeding education and support for caregivers. Finally, national and local programs should prioritize interventions that will improve food security and affordability especially in larger households.

### Limitations of the study

The study has some limitations that should be considered when interpreting the findings. First, the cross-sectional design limits the ability to establish causal associations between the identified factors and complementary feeding practices. Second, because the study relied on self-reported practices, there is the likelihood for a recall bias and social desirability bias especially for the 24-hour dietary recall and the timing of introduction of complementary foods. In addition, some sub-group analysis, particularly among unmarried mothers and caesarean deliveries, involved relatively small sample size which may have reduced estimate stability and may have effects on some odds ratio estimates, leading to wide confidence intervals. More so, member checking was not conducted for the qualitative findings due to logistical constraints. Finally, the qualitative findings are context-specific and may not be generalizable beyond similar socio-cultural settings.

## Conclusion

This study reveals that while timely initiation of complementary feeding is high, the rates of MDD, MMF, MAD, and appropriate complementary feeding practices remain suboptimal in Northern Ghana. These practices are found to be influenced by a wide range of socio-demographic factors such as ethnicity, maternal, child, and household characteristics, and antenatal care utilization. The qualitative findings also reveal the role of mothers-in-law and other family norms in shaping complementary feeding. Most of these family norms often undermine adherence to recommended WHO practices. The study further emphasizes the need for culturally sensitive and community-based nutrition interventions that will engage influential household members and strengthen infant and young child feeding counseling during antenatal and postnatal care. Finally, future studies should adopt longitudinal designs in order to provide better understanding of the causal pathways.

## Supplementary Information

Supplementary Material 1.

Supplementary Material 2.

## Data Availability

The datasets generated and/or analyzed during the current study are not publicly available due to ethical restrictions involving human participants but are available from the corresponding author on reasonable request, subject to approval by the relevant ethics committee.
