# Supplementary material for: Complementary feeding practices in Northern Ghana: a mixed-methods study of prevalence, predictors, and socio-cultural influences
Source: BMC Nutr. 2026 Jun 15;12:176. doi: 10.1186/s40795-026-01398-x (PMC13495485; doi:10.1186/s40795-026-01398-x)
Supplement: Supplementary file 1 [file 40795_2026_1398_MOESM1_ESM.docx]

**QUESTIONNAIRE FOR QUANTITATIVE DATA**

**TOPIC: ASSESSING THE ROLE OF SOCIOCULTURAL FACTORS IN CHILD FEEDING PRACTICES IN THE YENDI MUNICIPALITY**

**INFORMED CONSENT**

The main objective of the study is to investigate socio-demographic and cultural influences on child feeding practices in the Yendi Municipality of Ghana. You are assured that all responses provided would be strictly confidential and used for academic purposes only. Please, your anonymity is guaranteed and your participation in this study is voluntary; however, your decision to participate will be highly appreciated. Thank you for your cooperation.

May I begin the interview now? YES	NO

**Questionnaire for caregivers**

**SECTION A: SOCIO – DEMOGRAPHIC CHARACTERISTICS**

I) **Mother data**
1. Age of respondent (years):_______________________
2. Level of education of respondent:	A) None	B) Primary	C) Secondary/TVET	D) Tertiary
3. Marital status of child’s mother: A) Single	B) Married	C) Divorced	D) Widowed	E) Separated	F) Cohabitation	G) Others (Specify) …………
4. Ethnicity of child’s mother:	A) Dagomba	B) Kotokoli	C) Mamprusi	D) Nanumba	E) Konkomba	F) Others ………………….
5. How many people are in your household? ……………………………..
6. How many ANC visits did you have before you delivered the present child? ………………
II) **Child data**
7. Age of child (months): ………….
8. Gender of child:	A) Male	B) Female
9. Where was (name of child) delivered? A) Health facility [ ]	B) Home [ ]
10. What was the mode of delivery? A) Normal delivery	B) Cesarean section (SC)
11. What is the birth-order of child? Please specify……………………………

**SECTION B: COMPLEMENTARY FEEDING PRACTICES (24-hour dietary recall)**

1. **Is (child’s name) currently breastfeeding?**
   1. Yes
   2. No
2. **If ‘Yes’ to section B question 1, was child breastfed in the last 24 hours?**
   1. Yes
   2. No
3. **If ‘Yes’ to section B question 2, how many times was child breastfed in the last 24 hours?**

**____________________________**

4. **If ‘No’ to section B question 1, how many months did you breastfeed him/her?**

______________________________

5. **At what age (in months) of the child (name) did you start to give him/her liquid/semi-solid/solid foods (such as porridge, soup, mashed foods, or family foods)?**

_______________________(Hint: assist mother to quote time of feeding initiation in months)

6. **How many times was child fed in the last 24 hours?**

_______________________(Hint: mention about dinner, breakfast, launch, super, snacks etc.)

Please, mention all the foods and drinks that were eaten by (Name of child) over the past 24 hours whether at home or outside the home. (Hint: start with meal eaten at supper yesterday).

| ***Eating moment*** | ***Name of dish*** | ***Ingredients and Quantity*** |
| --- | --- | --- |
| Breakfast |  |  |
| Snack before lunch |  |  |
| Lunch |  |  |
| Snack before dinner |  |  |
| Dinner |  |  |
| Snack after dinner |  |  |
| Drinks |  |  |
| Any other food taken |  |  |

***Thank you for your time!!!***
