# Supplementary material for: Complementary feeding practices in Northern Ghana: a mixed-methods study of prevalence, predictors, and socio-cultural influences
Source: BMC Nutr. 2026 Jun 15;12:176. doi: 10.1186/s40795-026-01398-x (PMC13495485; doi:10.1186/s40795-026-01398-x)
Supplement: Supplementary file 2 [file 40795_2026_1398_MOESM2_ESM.docx]

**INTERVIEW GUIDE FOR QUALITATIVE**

**TOPIC: ASSESSING THE ROLE OF SOCIOCULTURAL FACTORS ON COMPLEMENTARY FEEDING PRACTICES IN THE YENDI MUNICIPALITY**

**INTRODUCTION**

The main objective of the study is to investigate socio-demographic and cultural influences on child feeding practices in the Yendi Municipality of Ghana. You are assured that all responses provided would be strictly confidential and used for academic purposes only. Please, your anonymity is guaranteed and your participation in this study is voluntary; however, your decision to participate will be highly appreciated. Thank you for your cooperation.

**Section A: Background Information of the Respondents**

1. Age of respondents
2. Parity
3. Religion
4. Ethnicity
5. Educational level
6. Occupation

**Section B: Beliefs about initiating complementary feeding**

1. When did you start feeding your child with liquid/semi-solid/solid foods? What was the reason for initiating feeding at this time?

***Probe:*** Was your decision to initiate feeding at this time influenced by someone or group of people (***hints:*** mother-in-laws, husbands, grandmothers etc.)

2. **What signs or indicators do you look for before deciding that a child is ready to start complementary feeding?**

**Probe:** How do you know that breast milk alone is no longer sufficient?

3. What are the perceived benefits or consequences of the timing of initiating complementary feeding?

***Probe:*** How do these timing decisions affect the child’s health, growth, or wellbeing?

**Section c: Beliefs and misconceptions against complementary feeding**

4. What foods are usually given to children during complementary feeding?

***Probe:*** what are the relevance of these foods mentioned to the child?

5. What are some cultural beliefs, taboos, superstitions, norms and traditions associated with complementary feeding?

***Probe:*** Are there foods children are not allowed to eat? Please explain why.

6. Do religious and/or community leaders play a role in deciding what foods children should eat?

***Probe:*** In what ways do they influence feeding practices within households or in the community?

7. Is there anything else you would like to share about complementary feeding practices in your community?

***Thank you for your time!!!***
